# Methods of massive parallel reporter assays for investigation of enhancers

**DOI:** 10.18699/VJ21.038

**Published:** 2021-05

**Authors:** S.E. Romanov, D.A. Kalashnikova, P.P. Laktionov

**Affiliations:** Novosibirsk State University, Epigenetics Laboratory, Department of Natural Sciences, Novosibirsk, Russia Institute of Molecular and Cellular Biology of the Siberian Branch of the Russian Academy of Sciences, Genomics Laboratory, Novosibirsk, Russia; Novosibirsk State University, Epigenetics Laboratory, Department of Natural Sciences, Novosibirsk, Russia Institute of Molecular and Cellular Biology of the Siberian Branch of the Russian Academy of Sciences, Genomics Laboratory, Novosibirsk, Russia; Novosibirsk State University, Epigenetics Laboratory, Department of Natural Sciences, Novosibirsk, Russia Institute of Molecular and Cellular Biology of the Siberian Branch of the Russian Academy of Sciences, Genomics Laboratory, Novosibirsk, Russia

**Keywords:** gene regulatory elements, enhancers, massive parallel assays, регуляторные элементы генома, энхансеры, высокопроизводительные методы анализа

## Abstract

The correct deployment of genetic programs for development and differentiation relies on finely coordinated regulation of specific gene sets. Genomic regulatory elements play an exceptional role in this process. There are few types of gene regulatory elements, including promoters, enhancers, insulators and silencers. Alterations of gene regulatory elements may cause various pathologies, including cancer, congenital disorders and autoimmune diseases. The development of high-throughput genomic assays has made it possible to significantly accelerate the accumulation of information about the characteristic epigenetic properties of regulatory elements. In combination with high-throughput studies focused on the genome-wide distribution of epigenetic marks, regulatory proteins and the spatial structure of chromatin, this significantly expands the understanding of the principles of epigenetic regulation of genes and allows potential regulatory elements to be searched for in silico. However, common experimental approaches used to study the local characteristics of chromatin have a number of technical limitations that may reduce the reliability of computational identification of genomic regulatory sequences. Taking into account the variability of the functions of epigenetic determinants and complex multicomponent regulation of genomic elements activity, their functional verification is often required. A plethora of methods have been developed to study the functional role of regulatory elements on the genome scale. Common experimental approaches for in silico identification of regulatory elements and their inherent technical limitations will be described. The present review is focused on original high-throughput methods of enhancer activity reporter analysis that are currently used to validate predicted regulatory elements and to perform de novo searches. The methods described allow assessing the functional role of the nucleotide sequence of a regulatory element, to determine its exact boundaries and to assess the influence of the local state of chromatin on the activity of enhancers and gene expression. These approaches have contributed substantially to the understanding of the fundamental principles of gene regulation.

## Abstract (ru)

Корректное развертывание генетических программ развития и дифференцировки опирается на тонко координированную регуляцию экспрессии специфических наборов генов. Исключительную роль в управлении этим процессом играют регуляторные элементы генома, к которым относятся промоторы, энхансеры, инсуляторы и сайленсеры. Нарушения в их работе могут приводить к развитию различных патологий, включая онкологические заболевания, пороки развития и аутоиммунные заболевания. Развитие технологий высокопроизводительного геномного анализа позволило значительно ускорить накопление информации о специфичных эпигенетических характеристиках регуляторных элементов. В совокупности с полногеномными исследованиями распределения эпигенетических меток, регуляторных белков и пространственной структуры хроматина такие данные значительно расширяют представления о принципах эпигенетической регуляции генов и позволяют осуществлять поиск потенциальных регуляторных элементов in silico. Вместе с тем основные экспериментальные подходы, используемые для исследования локальных характеристик хроматина, имеют ряд технических ограничений, которые снижают достоверность биоинформатической идентификации регуляторных областей генома. В связи с этим, а также с учетом вариабельности функций эпигенетических детерминант и многокомпонентной регуляции работы элементов генома определение их регуляторной роли часто требует функциональной проверки. Разработано множество методов, позволяющих провести исследование функциональной роли регуляторных элементов в масштабе генома. В настоящем обзоре кратко описаны основные экспериментальные подходы для проведения идентификации регуляторных элементов in silico и присущие им технические ограничения. Рассмотрены оригинальные методы высокопроизводительного репортерного анализа активности энхансеров, которые используют для валидации предсказанных регуляторных элементов и de novo поиска. Описанные методы анализа дают возможность оценить функциональную роль нуклеотидной последовательности регуляторного элемента, определить его точные границы, а также оценить влияние локального состояния хроматина на активность энхансеров и экспрессию генов. Применение таких методологических подходов обеспечило значительный вклад в понимание фундаментальных принципов регуляции генной экспрессии.

## Introduction

The progress of programs for the development and maintenance of body functions is based on the expression of gene sets specific to cells and tissues. The gene expression is coordinated by a multilevel regulatory system that includes genetic and epigenetic mechanisms based on the interaction of genomic sequences, epigenetic modifications, regulatory proteins, and specific transcription factors. Certain genomic regions associated with the specific epigenetic determinants, as well as serving as a site for attracting regulatory proteins, are capable of modifying gene expression. Such regulatory elements in the genome play a key role in the implementation of genetic programs for development, differentiation, and maintenance of cellular and tissue homeostasis (PhillipsCremins, Corces, 2013; Andersson et al., 2014; Kundaje et al., 2015).

Dysfunction of genomic regulatory elements may lead to the development of various pathologies, including cancer, developmental defects and autoimmune diseases (Maurano et al., 2012; Corradin et al., 2014; Miguel-Escalada et al., 2015; Bradner et al., 2017; Chatterjee, Ahituv, 2017 ). The genome wide association studies show that more than 90 % of disease-associated single nucleotide polymorphisms are located in non-coding genomic regions (Manolio et al., 2009; Maurano et al., 2012). The significant part of the genomic variants are located in regions that show epigenetic characteristics of enhancers, as well as affect enhancers, specific for the cell lines involved in the disease pathogenesis (Ernst et al., 2011; Akhtar-Zaidi et al., 2012; Trynka et al., 2013). The genetic variants associated with the development of type 2 diabetes (T2D), which were located in regions of putative enhancers in pancreatic islets, can be a good example (Stitzel et al., 2010; Pasquali et al., 2014). 

Today, a lot of information is available regarding the specific properties of the epigenetic regulatory elements that alleviate identification of potential regulatory genomic regions in silico (Ernst et al., 2011). However, the validation and functional characterization of the regulatory elements often requires direct experimental verification. The classic methods are different modifications of reporter assays and functional mutagenesis. With the development of massively parallel sequencing methods, methodologies that allow studying the activity of the regulatory elements in genomescale have been developed. 

This review will describe the existing methodological solutions in the high-throughput analysis of enhancers that have significantly contributed to the understanding of the fundamental principles of their functions.

## Types of regulatory elements

Several types of genomic regulatory elements, including promoters, enhancers, insulators, and silencers are distinguished. 

Promoters are located near the transcription start site and serve as a DNA site where the transcription complex is assembled. In eukaryotes, such transcription complexes consist of the main transcription factors, RNA polymerase, and other regulatory proteins, including those which mediate the interaction with enhancers (Andersson, Sandelin, 2020). 

Enhancers are nucleotide sequences in genomic DNA that contain binding sites for transcription factors and cofactors. As part of a protein complex, enhancers can physically interact with the promoter to activate gene expression (Shlyueva et al., 2014). Enhancers are able to regulate target promoters from a long distance, and regardless of mutual spatial orientation (Pennacchio et al., 2013). For example, the ZRS enhancer, the dominant mutation of which leads to familial forms of polydactyly, is located approximately 1 Mb from the controlled Sonic hedgehog (Shh) gene in the mouse genome (Lettice et al., 2014). On average, the enhancers are mapped at 20–50 Kb from the target gene in vertebrate genomes, and at 4–10 Kb in the genome of the fruit fly (Furlong, Levine, 2018).

The regulatory interactions network of promoters and enhancers can be quite complex. A separate gene can share enhancers with other genes, might be regulated either by several enhancers or specific enhancers in different types of cells. The Arx gene expression, for example, is controlled by four enhancers in mouse brain tissue (Dickel et al., 2018). Regulation of a gene by specific enhancers is also observed during the development of pathologies. For example, the Myc proto-oncogene enhancer is located in transcription termination sites in case of pancreatic cancer. In case of rectal cancer, it is detected in the 5′-region of the gene, and in case of T-cell acute lymphoblastic leukemia, it can be found downstream of the 3′-region of the gene (Sur, Taipale, 2016). 

Studies conducted in Drosophila melanogaster have shown that up to 30 percent of enhancers can act as remote regulatory elements without affecting the expression of genes located between them and target genes (Ghavi-Helm et al., 2014; Kvon et al., 2014). This means that there must be fine-tuned regulatory mechanisms that address interaction between the target gene promoter and specific enhancer. Today, there are several functionally intersecting concepts describing mechanics of the promoter-enhancer interactions, the main of which are contact formation via protein homooligomers and chromatin looping, caused by the action of motor proteins, such as RNA polymerase II and cohesin. 

The regulatory elements – insulators – play an important role in regulation of the chromatin spatial structure. Interacting with specific proteins, insulators are able to block enhancer-promoter interaction and prevent the distribution of repressive chromatin marks acting as barrier elements (Kellum, Schedl, 1991, 1992; Geyer, Corces, 1992; Cai, Levine, 1995). With the development of modern methods of the nuclear architecture analysis, it became apparent that the functional impact of insulators is largely determined by their participation in the regulation of intra- and interchromosomal contacts (Yang, Corces, 2011). The insulator proteins play a key role in the formation of topologically associated domain (TAD) (Dixon et al., 2012). Such fragments are characterized by a high frequency of internal DNA contacts and are often flanked by the binding sites of insulator proteins and actively transcribed genes (PhillipsCremins et al., 2013; Rao et al., 2014). Along with the regulation of the nucleus spatial structure, insulators are involved in many regulatory processes, including activation and repression of the gene expression, alternative splicing, and RNA polymerase pausing (Shukla et al., 2011; Paredes et al., 2013; Phillips-Cremins, Corces, 2013). 

The silencers function is to suppress the gene expression, and such repression is mainly implemented by establishing repressive chromatin state and competition with activating proteins (Li et al., 2004; Srinivasan, Atchison, 2004; Harris et al., 2005; Lanzuolo et al., 2007; Tiwari et al., 2008).

## Identification of regulatory genomic elements

The development of modern methods of high-throughput analysis has significantly accelerated and simplified the search for potential regulatory elements. The assumptions about the possible regulatory role of a genomic region are usually based on several types of data, including: (1) DNA accessibility for regulatory proteins, (2) presence of characteristic epigenetic determinants, (3) evaluation of gene expression and (4) analysis of DNA contacts. 

Active regulatory elements are associated with specific proteins, and, hence, are free from nucleosomes. The treatment of genomic DNA with DNase I (DNase-seq), micrococcal nuclease (MNase-seq) and Tn5 transposase (assay for transposase-accessible chromatin, ATAC-seq), followed by high throughput sequencing and FAIRE-seq method, is used to identify such nucleosome-free loci (Nagy et al., 2003; Gaulton et al., 2010; Song, Crawford, 2010; Buenrostro et al., 2013). The listed methods are used for identification of putative enhancers, insulators, and silencers; however, to determine functional class of detected regulatory element, data on DNA accessibility should be combined with other descriptive data, e.g. chromatin properties (Song et al., 2011; Murtha et al., 2014; Huang et al., 2019).

The genomic mapping of the chromatin characteristic factors and histone modifications is also used to identify individual classes of regulatory elements. The basic method for assessing the representation of such epigenetic determinants in a particular genomic region is the chromatin immunoprecipitation followed by massive parallel sequencing (ChIP-seq). Promoters are enriched in H3K4me3 histone mark (Bernstein et al., 2005). Monomethylation at the same position of the H3 histone (H3K4me1) is associated with enhancers, and the simultaneous presence of the H3K27me3 modification indicates that the enhancer might be poised for activation, while the H3K27ac modification indicates that the enhancer is active (Heintzman et al., 2007; Creyghton et al., 2010; Rada-Iglesias et al., 2011; Bonn et al., 2012; Arnold et al., 2013). Enrichment in the p300 histone acetyltransferase is characteristic of the enhancers (Visel et al., 2009). Mapping of specific transcription factors is also used to identify enhancers. For example, DNA regions enriched by the active enhancer histone marks, the Mediator complex proteins and the Oct4, Sox2, Nanog, Klf4, Esrrb master regulators are called super-enhancers and control the expression of tissuespecific sets of genes in embryonic stem cells (Whyte et al., 2013). To identify insulators in vertebrates, the genomic distribution of the CTCF protein and cofactors involved in the formation of loops, such as Rad21 and YY, are analyzed (Dixon et al., 2012, 2015; Nora et al., 2017; Rao et al., 2017). Silencers are enriched by the H3K27me3 histone modification associated with the effect of repressive Polycomb group proteins, as well as the H3K9me2/3 modifications related to heterochromatin (Barski et al., 2007).

The spatial organization of the nucleus mediates the interactions between target genomic loci and distal regulatory elements. The spatial chromatin structure is studied by methods that allow to fix and analyze DNA-DNA contacts, which originate from the 3C method (chromosome conformation capture) (Dekker et al., 2002; Tolhuis et al., 2002). The most widely used HiC method allows to build DNA genome-wide contacts map, in contrast to earlier methods (Gavrilov et al., 2009; Lieberman-Aiden et al., 2009). Combinations of chromatin spatial structure analysis and chromatin immunoprecipitation-based methods (ChIA-PET, HiChIP, and PLAC-ChIP) make it possible to establish DNA contacts in genomic regions which are specifically enriched in specific chromatin proteins or histone modifications (Fullwood, Ruan, 2009; Fang et al., 2016; Mumbach et al., 2016). Analysis of the DNA-DNA contacts allows identifying promoter-enhancer interactions, defining borders of the topologically associating domain and larger chromatin compartments.

The data on the epigenetic characteristics and spatial genomic organization of model objects are available to a wide range of researchers in ENCODE, The Epigenome Roadmap, FANTOM and other databases (Birney et al., 2007; Bernstein et al., 2010; Andersson et al., 2014; Forrest et al., 2014; Kellis et al., 2014; Kundaje et al., 2015). These data are widely used for the prediction and research of potential regulatory elements. 

However, it must be noted that the methods of analysis of protein-DNA and DNA-DNA interactions are capable of detecting non-functional interaction that can result in a false positive result. Local enrichment with characteristic epigenetic determinants detected by ChIP-seq does not necessarily indicate the presence of regulatory elements in a specific genomic region (Kvon et al., 2012). This can be due to the fact that implementation of the regulatory element function might require the coordinated binding of several transcription factors, and binding of only one of them is simply not enough (Halfon et al., 2000; Sandmann et al., 2007). 

Nonfunctional transcription factor binding events can be transient, and caused by the general DNA-binding activity (Hammar et al., 2012). The chromatin immunoprecipitation method detects such transient interactions since it is based on the fixation of chromatin with formaldehyde with the formation of covalent cross-links between DNA and associated proteins. Modifications of the ChIP method that eliminate the need for chromatin fixation and potentially improve the accuracy of the method have been proposed (Skene, Henikoff, 2017; Kaya-Okur et al., 2019). A micrococcal nuclease fused with protein A is used in the variation of the CUT&RUN method (Skene, Henikoff, 2017). Protein A binds with the specific antibodies to the target protein, and micrococcal nuclease makes DNA breaks in the region of its binding. This allows selecting short genomic fragments, which are rich in proteins of interest, and identifying them with high-throughput sequencing. The Tn5 transposase is used in the CUT&TAG method instead of nuclease, which makes it possible to simultaneously introduce DNA adapters for massive parallel sequencing, flanking the recognition site of the protein of interest (Kaya-Okur et al., 2019). However, these methods have been developed recently and have not been widely used yet.

The false positive results in the ChIP-seq experiments may also be due to experimental variations, such as chromatin fragmentation mode, sequencing depth, and the threshold values for the identification of binding sites (Rye et al., 2011; Gomes et al., 2014; Jung et al., 2014). It is also important to note that in the presence of high- and low-affinity protein binding sites, the ChIP-seq method predominantly detects high-affinity ones (Nettling et al., 2016). This feature is also a limitation of the method, since it was shown that suboptimal binding sites for transcription factors in enhancers are needed for fine regulation of gene activity during development (Crocker et al., 2015, 2016; Farley et al., 2015). 

In addition to the technical limitations of experimental methods, it is important to note that often functional regulatory elements demonstrate the presence of epigenetic determinants, which is generally uncharacteristic for their class. Functionally tested silencers in the K562 and HepG2B cell cultures, according to the ENCODE database, in addition to being enriched with the H3K9me3 and H3K27me3 repressive histone modifications, also contained H3K36me3 and H3K79me2 active chromatin histone marks (Pang, Snyder, 2020). Due to the experimental limitations of the methods, the variability of the functions of epigenetic determinants, and the participation of many components in the implementation of the functions of genomic elements, the determination of their regulatory role often requires functional verification. 

## Enhancer research methods

The functional role of genomic regulatory elements is commonly assessed with different modifications of reporter analysis. Pioneer work where the functional role of the genomic regulatory elements was demonstrated was devoted to the study of the enhancer of the early gene of the SV40 virus (Banerji et al., 1981). This work showed that a DNA fragment from the 5′-end of the early gene of the SV40 virus, consisting of two 72 bp repeats, can cause 200-fold activation of rabbit β-globin reporter gene expression in the HeLa cells (Banerji et al., 1981). 

The standard genetic constructs used for the analysis of enhancer activity contain a reporter gene under the control of a minimal promoter, which confers minimal or no expression without additional activation. The genomic sequence of the enhancer is cloned into the construct, either upstream of the promoter or downstream of the coding sequence of the reporter gene. The obtained construct is used to transform cells and the change in the expression of the reporter gene comparing to a control construct that does not contain a potential enhancer is analyzed. 

One of the first works aimed at in vivo functional testing of enhancers in genome-wide scale was based on the principles of classical reporter analysis (Kvon et al., 2014). Around 8,000 of the D. melanogaster lines were used, which contained a transgenic construct consisting of potential enhancer, minimal promoter, and Gal4 protein gene integrated in the same genomic region. The Gal4 expression was assessed at different stages of embryogenesis by in situ hybridization, and 400 embryos at different stages of development were analyzed for each potential regulatory element. As a result, more than three thousand enhancers have been identified. About a quarter were located in the vicinity of regulated genes, and a little more than a quarter were located at a distance of 20–100 Kb. On average, they were mapped around 10 Kb from the target genes (Kvon et al., 2014). About one third of the detected enhancers were located in the intergenic regions of regulated genes. Subsequently, it was also functionally confirmed that enhancers are able to regulate not only nearby genes, but also ones located through one or two genes (Kvon et al., 2014). The data obtained have significantly expanded the understanding of the fundamental principles of the operation of enhancers; however, the implementation of such projects requires a colossal amount of time and resources. 

## High-throughput enhancer reporter assays

**Fig. 1.**
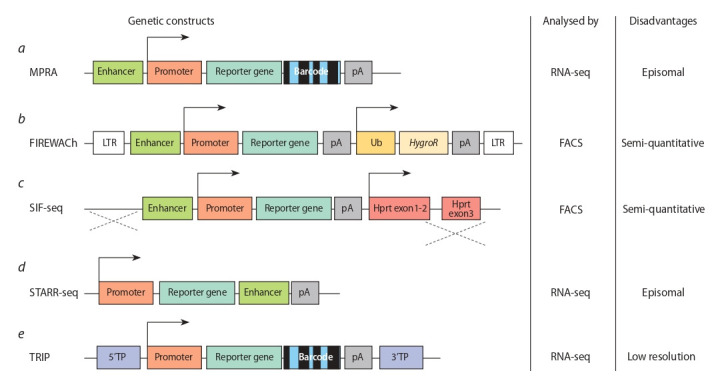
Genetic constructs used for MPRA (a–d) and TRIP method (e). pA – the polyadenylation signal; LTR – long terminal repeat; Ub – ubiquitin promoter; HygroR – hygromycin resistance gene; Hprt exon – the Hprt gene exon.

**Fig. 2.**
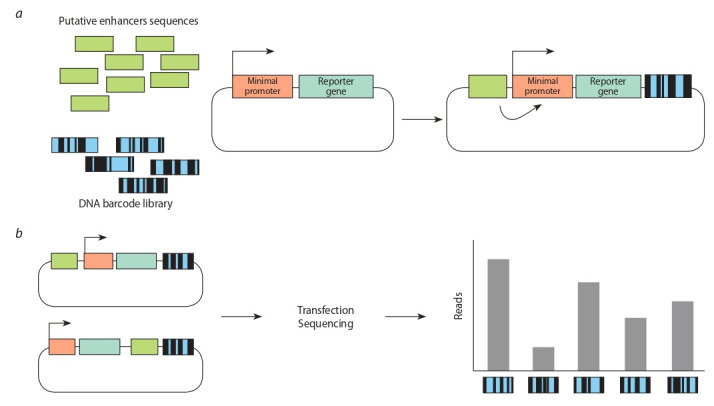
Enhancer testing with MPRA. a – at the first stage of MPRA, a pool of potential regulatory sequences is prepared. To obtain such sequences, synthesis technologies are used, or enrichment by chromatin immunoprecipitation methods, etc. Then, a pool of genetic constructs containing a minimal promoter and a reporter gene is created under the control of the putative regulatory elements. Each regulatory element in such constructs is associated with unique DNA barcode located at the end of the coding sequence of the reporter gene; b – upon transfection of cells, the expression of the reporter gene is activated in case putative regulatory element exhibits enhancer properties. The RNA-seq method is used to assess the expression level of unique DNA barcodes in the cell transcriptome. Normalization for barcode representation in initial pool and defining DNA fragments corresponding to each unique barcode allows to determine enhancer activity of tested genome region.

The methods of high-throughput reporter analysis have evolved from classical approaches and allow simultaneous interrogation of thousands of regulatory sequences. There are two principal approaches in high-throughput reporter assays (Fig. 1). Within the first, a reporter gene contains a DNA barcode before the polyadenylation signal, and is placed under the control of a genomic fragment – a potential enhancer and a minimal promoter (see Fig. 1, a). In the case of activation of the reporter gene expression, such DNA barcode will be contained at the 3′-end of its transcript. After the pooling of such constructs, high-throughput sequencing is carried out, and unique DNA barcodes corresponding to each of the studied genomic fragments are determined (Fig. 2). After transformation using such constructs, the presence of DNA barcodes is analyzed by the transcriptome sequencing (RNA-seq). The expression level of a particular DNA barcode allows to assess activating ability of the corresponding specific regulatory element. This approach is used in the methods of quantitative assessment of the activity of genomic fragments, called MPRA (massive parallel reporter assay), different variations of which will be covered in this review (Kwasnieski et al., 2012, 2014; Melnikov et al., 2012; Kheradpour et al., 2013; Maricque et al., 2017).

The second approach allows evaluating the qualitative ability of the genomic fragment to exhibit the enhancers’ properties. At first, a pool of genetic constructs that contain the genomic fragment of interest, a minimal promoter, and a reporter gene encoding a fluorescent protein or luciferase is prepared. At the next stage, the obtained pool of constructs is used for transformation and cells expressing the fluorescent protein are sorted using flow cytometry. The activation of the reporter gene expression means that the genomic fragment of interest demonstrates enhancer activities. The sorted cells are subjected to DNA isolation, fragments of constructs corresponding to the studied genomic fragments are amplified, and massive parallel sequencing is carried out, thus allowing to identify specific genomic fragments exhibiting the properties of enhancers. Examples of such methods include FIREWACh and SIF-seq (Dickel et al., 2014; Murtha et al., 2014) (see Fig. 1, b, c). 

Combinations of the two approaches described above are also used. In this case, at the first stage cells carrying constructs containing potential enhancers are selected by flow cytometry. Then, the activating ability of specific genomic fragments is quantified by analyzing the representation of DNA barcodes by the RNA-seq method (Maricque et al., 2018). 

MPRA methods are successfully used to study the activating properties of the nucleotide sequence of enhancers, the functional influence of regulatory protein binding motifs, and to search for and validate enhancers. Using this methodology, the effect of single mutations in the composition of three enhancers – ALDOB, ECR11, and LTV1, active in liver cells, was studied (Patwardhan et al., 2012). In the course of this research, a DNA library containing more than 100,000 mutated variants of the enhancers was synthesized. Such DNA fragments were cloned into constructs containing the minimal promoter, luciferase gene and transcribed DNA barcodes. The resulting DNA libraries were injected into the liver of mice, and a day later the transcriptome of liver cells was analyzed by RNA-seq (Kim, Ahituv, 2013).

It was found that the majority of single mutations had a weak effect on the activity of the studied enhancers. In addition, it was shown that mutations disrupting the enhancer function affect the predicted binding sites of the HNF4 and HNF1 transcription factors, which are active in liver cells (Kel et al., 2003). It is important to note that the experiment also showed significant discrepancies in theory and practice. Thus, within the ECR11 enhancer, mutations causing functional disorders were concentrated in the region that did not contain predicted binding sites for transcription factors, while mutations in the region containing most of these predicted sites did not change the enhancer activity. On the one hand, this clearly demonstrates that MPRA are applicable to clarify the boundaries of enhancers, and on the other hand, it emphasizes the importance of experimental verification of predictive data.

MPRA are also used for de novo search and validation of predicted enhancers. An elegant approach to search for enhancers was implemented in the STARR-seq method (Arnold et al., 2013) (see Fig. 1, d ). The authors used the ability of enhancers to activate expression regardless of the position relative to the gene and promoter, and developed reporter constructs, containing studied genomic fragments cloned into open reading frame downstream from minimal promoter. In case a genomic region exhibits an enhancer function, this will lead to its transcription in cells. The expression level of this fragment in the cell transcriptome makes it possible to assess its activating function. This approach completely eliminates the need to use DNA barcodes, since the fragments play that role themselves. 

To seek for enhancers, a plasmid library containing millions of random fragments of the fruit fly genome was prepared. After transfection of the S2 cell culture, a highthroughput RNA-seq transcription profile analysis was performed. As a result, thousands of genomic fragments were identified, which demonstrated the properties of enhancers. The most active were located close to housekeeping genes and developmental transcription factors. About a third of the fragments demonstrating pronounced activating properties in the S2 cell genome were located in areas of closed chromatin, lacking H3K27ac mark of active enhancers. Thus, it seems unlikely that such fragments are capable of performing the role of enhancers in the genome of the studied cells, and this finding highlights some of the limitations of episomal MPRA, which will be discussed below.

An interesting modification of the STARR-seq method was used in a subsequent work on the study of enhancers in human embryonic stem cells (Barakat et al., 2018). In the original work, DNA libraries were obtained by ultrasonic fragmentation of the D. melanogaster genomic DNA and subsequent mass cloning of the obtained fragments (Arnold et al., 2013). However, this approach is poorly applicable to larger genomes, since a sufficient representation of regulatory elements in the resulting DNA libraries is an extremely difficult task to achieve. Indeed, the use of the original STARR-seq method to study the regulatory elements of the mouse genome will require the creation of more than 200 million unique constructs (Murtha et al., 2014). Experimental verification showed that the use of a plasmid library containing 1.3 million unique fragments of the human genome made it possible to identify only six enhancers (Murtha et al., 2014).

To overcome this limitation, the ChIP-STARR-seq method was proposed. In the original paper, the chromatin immunoprecipitation was used to isolate genomic fragments enriched with the OCT4, NANOG transcription factors, as well as the H3K4me1 and H3K27ac histone modifications (Barakat et al., 2018). Obtained DNA fragments were then cloned into DNA libraries similar to those used in the original method. It was found that only a part of the genomic fragments that demonstrate enrichment by these factors in genome exhibited enhancer activity. Only about 25 % of the fragments bound by OCT4 showed enhancer properties. For the fragments enriched in NANOG and the H3K4me1 and H3K27 histone modifications, the results were 15, 9, and 10 %, respectively. It has been shown that neither individual factors nor their combinations are capable of unambiguously predicting enhancers. In addition, a group of enhancers associated with the regulation of general cellular processes, which had not previously been found in ESCs, were found. It turned out that such enhancers demonstrate a rather weak enrichment in TF OCT4 and NANOG, as well as in the histone modification H3K4me1, and, most likely, for this reason they were not previously detected in prospecting studies based on the chromatin immunoprecipitation method. 

Data on chromatin accessibility and the genomic distribution of histone modifications and regulatory proteins deposited in open repositories allow to predict regulatory elements. Using MPRA, the activity of regulatory elements in the K562 cells and the E1 human embryonic stem cells, identified on the basis of chromatin structure analysis and annotated in ENCODE, was studied (Kwasnieski et al., 2014). It turned out that only about a quarter of them had an effect on gene expression, which underlines the importance of such experimental verification (Kwasnieski et al., 2014). At the same time, this effect may be due to the experimental limitations of the described MPRA. Indeed, the methods described above are episomal, which means that reporter constructs are not integrated into the genome, hence the activity of enhancers is assessed outside the chromatin context. Significant differences in the activity of enhancers analyzed in episomal manner and upon integration into the genome were also confirmed experimentally (Inoue et al., 2017).

This experimental discrepancy looks logical, because the observation of the effect of chromatin structure on gene regulation was demonstrated in classical genetic experiments long ago (Muller, 1930). The use of an original high-throughput reporter analysis method, combined with MPRA-approaches, made it possible to characterize the local effects of chromatin on gene expression in mouse embryonic stem cells (Akhtar et al., 2013) (see Fig. 1, e). Within the framework of this study, using the PiggyBac transposasebased genomic integration system, reporter constructs containing unique DNA barcodes at the 3′-end of the reporter gene were randomly inserted into the cell genome.

In the next step, such insertions were mapped and each DNA barcode was associated with a specific genomic locus. In total, more than 17 thousand of such inserts were received. Then, the expression level of DNA barcodes was analyzed using the RNA-seq method, which made it possible to assess the transcriptional activity of each insertion as well as the effect of the local chromatin structure on it. Reporter constructs integrated into regions of compacted chromatin and regions of domains associated with the nuclear lamina, as expected, showed a reduced level of expression. Reporter constructions located within 200 Kb from active genes were more actively transcribed. It is interesting to mention that an increased frequency of enhancers was observed within approximately the same range. Enhancers had an activating effect on the expression of reporter constructs at a distance of up to 20 Kb. It is important to note that in this case a linear distance is considered, and the spatial structure of chromatin is not taken into account. It was assumed that the formation of extended, actively transcribed regions is based on the action of several enhancers. This emphasizes the need to study regulatory elements in conditions close to native ones.

The effect of chromatin on the function of regulatory elements is to some extent taken into account in MPRA, which are based on the genomic integration of the reporter construct (Dickel et al., 2014; Murtha et al., 2014; Maricque et al., 2017, 2018). These FIREWACh and SIF-seq methods were used to identify enhancers in mouse ESCs, but did not allow quantitative assessment of the activity of regulatory elements (see the general description of approaches above) (Dickel et al., 2014; Murtha et al., 2014).

The FIREWACh method is based on genomic integration of reporter constructs using lentiviral transduction (see Fig. 1, b). This method of genomic integration ensures the insertion of the construct into random regions of the genome (Yang et al., 2008). Thus, an adequate comparison of the activity of various regulatory elements seems to be difficult, because it is highly likely that reporter constructs will be integrated into different genomic regions, with an unpredictable effect of the local chromatin environment. 

The SIF-seq method avoided such a drawback, since the integration of reporter constructs is carried out in the same region of the genome located in the region of the Hprt gene (Dickel et al., 2014) (see Fig. 1, b). However, this might serve as a disadvantage, since the correct operation of an enhancer is determined by a specific set of chromatin factors, and it is highly likely that it will become non-functional when transferred to a non-identical chromatin environment.

The approaches described above did not allow answering one of the fundamental questions of understanding the principles of enhancers’ activity, namely, to what extent is it determined by the DNA sequence, and to what extent – by the properties of the surrounding chromatin? A systematic study of this issue was carried out in the research on the influence of different chromatin environments on the comparative activity of enhancers (Maricque et al., 2018). Within the framework of this study, 15 lines of the K562 cells were prepared, containing single insertions of reporter constructs located in different chromatin environments and containing the Cre-recombinase (loxP) recognition sites, allowing targeted insertion of transgenes. Such insertions contained a DNA barcode and a polyadenylation signal outside of the fragment flanked by loxP-sites, with a single unique DNA barcode corresponding to each line.

The described lines were pooled together, and Cre-mediated integration was used to integrate reporter constructs, that contained a reporter gene ending with a DNA barcode under the control of the minimal promoter and the genomic fragment of interest. As such fragments, 300 synthesized regulatory elements were used, which were previously studied by episomal MPRA and ranked according to the level of activity (Kwasnieski et al., 2014). For each genomic fragment, the corresponding unique DNA barcodes had been previously established. In case of successful integration, the original loxP cassette was replaced with a reporter construct containing the putative enhancers. Moreover, in the case of activation of the reporter gene, two DNA barcodes will be transcribed in its composition. Deciphering barcodes allows to identify which fragment was analyzed in which cell line.

The analysis of the representation of combinations of DNA barcodes in the transcriptome of cells made it possible to assess the level of activity of the studied regulatory elements in different chromatin environments. It was found that the chromatin environment has pronounced effect on the activity of cis-elements. However, being placed in the same chromatin environment, regulatory elements save their relative activity. It was also demonstrated that the activity of the promoter affects the expression of reporter constructs, but at the same time does not affect the comparative activity of regulatory elements. The results obtained support the model according to which the nucleotide sequence of the enhancer determines its overall activity, which is already modulated by the structure of the chromatin environment. 

## Conclusion

MPRA methods allow to perform detailed study of the regulatory potential of the genomic fragments, and it is a convenient tool for studying the effect of variations in the nucleotide sequence on their function. However, it is necessary to note the limitations of the methods, which should be taken into account when interpreting the results obtained. The common drawback of MPRA is the need to use a minimal promoter that is unable to activate the expression of the reporter gene in the absence of an enhancer, since the presence of basal activity can significantly distort the results. At the same time, the selected promoter can significantly influence the activity of a particular enhancer ( Zabidi et al., 2015; Maricque et al., 2018). In this sense, the analysis of the activity of enhancers in combination with various promoters seems to be an ideal experiment. However, such work seems to be extremely difficult and time-consuming

The synthesis of DNA fragments used as studied regulatory elements imposes restrictions on the total length of such a fragment. Usually, the length of the studied fragments is limited to about 200 bp, which often complicates the analysis of the influence of the rest of the enhancer regions falling outside these limits (Kwasnieski et al., 2014). MPRA methods based on episomal constructs do not take into account the possible influence of the chromatin environment on the regulatory element; therefore, they can be used to study the direct activating ability of a DNA sequence. MPRA based on the genomic integration of reporter constructs make it possible to overcome this limitation to some extent. However, random or site-specific integration still does not allow the analysis of the activity of a regulatory element in native genomic environment. The impossibility of studying the enhancers function in native environment is a serious MPRA limitation, since the function of the regulatory genomic element depends on the structure of the surrounding chromatin and the spatial organization of the locus.

Modern methods of high-throughput CRISPR/Cas9 mutagenesis, as well as methods of directed expression modulation based on the use of an inactivated form of the Cas9 endonuclease (dCas9) fused with activator or repressor proteins, make it possible to study regulatory elements in native genomic environment (Chavez et al., 2015; Sanjana et al., 2016; Canver et al., 2017; Li et al., 2020). While there are obvious advantages, such methods also have potential drawbacks. For example, point mutations produced by the targeted mutagenesis may not be sufficient to disrupt enhancer function. In addition, directed mutagenesis is associated with errors in the recognition of target genome regions (off-targets), which can lead to the generation of experimental noise. 

It is important to note that the KRAB repressor protein, which is widely used for the targeted inactivation of enhancers, is capable of initiating the formation of heterochromatin regions of 1–2 Kb in length (Gasperini et al., 2019). This feature can reduce the resolution of the method and complicate the identification of specific functional fragments of the enhancer, as well as increase unwanted side effects in the case of the presence of erroneous dCas9 recognition sites. In addition to possible technical difficulties, in the case of a successful disruption of the enhancer function, phenotypic manifestations can be restored rather quickly due to the presumable existence of duplicate enhancers (Diao et al., 2016).

Thus, MPRA and high-performance methods based on the CRISPR/Cas9 system are quite complementary and make it possible to characterize in detail the regulatory functions of the studied genomic fragments. Coupled with vast amounts of accumulated data on the chromatin structure and spatial organization in various cells and tissues, the use of such methods makes it possible to significantly advance in the understanding of the mechanisms of precise regulation of gene expression during development and in various pathologies. Altogether, this allows hoping that in the near future modern genomics will be able to move from a detailed functional description of regulatory elements to the creation of quantitative biological models for the regulation of gene expression.

## Conflict of interest

The authors declare no conflict of interest.

## References

Akhtar-Zaidi B., Cowper-Sal-lari R., Corradin O., Saiakhova A., Bartels C.F., Balasubramanian D., Myeroff L., Lutterbaugh J., Jarrar A.,
Kalady M.F., Willis J., Moore J.H., Tesar P.J., Laframboise T., Markowitz S., Lupien M., Scacheri P.C. Epigenomic enhancer prof iling
defines a signature of colon cancer. Science. 2012;336:736-739.

Akhtar W., de Jong J., Pindyurin A.V., Pagie L., Meuleman W., de Ridder J., Berns A., Wessels L.F., van Lohuizen M., van Steensel B.
Chromatin position effects assayed by thousands of reporters integrated in parallel. Cell. 2013;154:914-927.

Andersson R., Gebhard C., Miguel-Escalada I., Hoof I., Bornholdt J.,
Boyd M., Chen Y., …, Suzuki H., Hayashizaki Y., Muller F., Forrest A.R.R., Carninci P., Rehli M., Sandelin A. An atlas of active
enhancers across human cell types and tissues. Nature. 2014;507:
455-461.

Andersson R., Sandelin A. Determinants of enhancer and promoter activities of regulatory elements. Nat. Rev. Genet. 2020;21:71-87.
Arnold C.D., Gerlach D., Stelzer C., Boryn L.M., Rath M., Stark A.
Genome-wide quantitative enhancer activity maps identified by
STARR-seq. Science. 2013;339:1074-1077.

Banerji J., Rusconi S., Schaffner W. Expression of a beta-globin gene
is enhanced by remote SV40 DNA sequences. Cell. 1981;27:299-
308.

Barakat T.S., Halbritter F., Zhang M., Rendeiro A.F., Perenthaler E.,
Bock C., Chambers I. Functional dissection of the enhancer repertoire in human embryonic stem cells. Cell Stem Cell. 2018;23:276-
288 e278.

Barski A., Cuddapah S., Cui K., Roh T.Y., Schones D.E., Wang Z.,
Wei G., Chepelev I., Zhao K. High-resolution profiling of histone
methylations in the human genome. Cell. 2007;129:823-837.

Bernstein B.E., Kamal M., Lindblad-Toh K., Bekiranov S., Bailey D.K.,
Huebert D.J., McMahon S., Karlsson E.K., Kulbokas E.J. 3rd, Gingeras T.R., Schreiber S.L., Lander E.S. Genomic maps and comparative analysis of histone modifications in human and mouse. Cell.
2005;120:169-181.

Bernstein B.E., Stamatoyannopoulos J.A., Costello J.F., Ren B., Milosavljevic A., Meissner A., Kellis M., Marra M.A., Beaudet A.L.,
Ecker J.R., Farnham P.J., Hirst M., Lander E.S., Mikkelsen T.S.,
Thomson J.A. The NIH roadmap epigenomics mapping consortium.
Nat. Biotechnol. 2010;28:1045-1048.

Birney E., Stamatoyannopoulos J.A., Dutta A., Guigo R., Gingeras T.R., Margulies E.H., Weng Z., …, Lander E.S., Koriabine M.,
Nefedov M., Osoegawa K., Yoshinaga Y., Zhu B., de Jong P.J.
Identification and analysis of functional elements in 1 % of the
human genome by the ENCODE pilot project. Nature. 2007;447:
799-816.

Bonn S., Zinzen R.P., Girardot C., Gustafson E.H., Perez-Gonzalez A., Delhomme N., Ghavi-Helm Y., Wilczynski B., Riddell A.,
Furlong E.E. Tissue-specific analysis of chromatin state identifies
temporal signatures of enhancer activity during embryonic development. Nat. Genet. 2012;44:148-156.

Bradner J.E., Hnisz D., Young R.A. Transcriptional addiction in cancer.
Cell. 2017;168:629-643.

Buenrostro J.D., Giresi P.G., Zaba L.C., Chang H.Y., Greenleaf W.J.
Transposition of native chromatin for fast and sensitive epigenomic
profiling of open chromatin, DNA-binding proteins and nucleosome
position. Nat. Methods. 2013;10:1213-1218.

Cai H., Levine M. Modulation of enhancer-promoter interactions by
insulators in the Drosophila embryo. Nature. 1995;376:533-536.

Canver M.C., Bauer D.E., Orkin S.H. Functional interrogation of noncoding DNA through CRISPR genome editing. Methods. 2017;
(121-122):118-129.

Chatterjee S., Ahituv N. Gene regulatory elements, major drivers of
human disease. Annu. Rev. Genomics Hum. Genet. 2017;18:45-63.

Chavez A., Scheiman J., Vora S., Pruitt B.W., Tuttle M., Eswar P.R.I.,
Lin S., Kiani S., Guzman C.D., Wiegand D.J., Ter-Ovanesyan D.,
Braff J.L., Davidsohn N., Housden B.E., Perrimon N., Weiss R.,
Aach J., Collins J.J., Church G.M. Highly efficient Cas9-mediated
transcriptional programming. Nat. Methods. 2015;12:326-328.

Corradin O., Saiakhova A., Akhtar-Zaidi B., Myeroff L., Willis J., Cowper-Sallari R., Lupien M., Markowitz S., Scacheri P.C. Combinatorial effects of multiple enhancer variants in linkage disequilibrium
dictate levels of gene expression to confer susceptibility to common
traits. Genome Res. 2014;24:1-13.

Creyghton M.P., Cheng A.W., Welstead G.G., Kooistra T., Carey B.W.,
Steine E.J., Hanna J., Lodato M.A., Frampton G.M., Sharp P.A.,
Boyer L.A., Young R.A., Jaenisch R. Histone H3K27ac separates
active from poised enhancers and predicts developmental state.
Proc. Natl. Acad. Sci. USA. 2010;107:21931-21936.

Crocker J., Abe N., Rinaldi L., McGregor A.P., Frankel N., Wang S.,
Alsawadi A., Valenti P., Plaza S., Payre F., Mann R.S., Stern D.L.
Low affinity binding site clusters confer hox specificity and regulatory robustness. Cell. 2015;160:191-203.

Crocker J., Noon E.P., Stern D.L. The soft touch: low-affinity transcription factor binding sites in development and evolution. Curr. Top.
Dev. Biol. 2016;117:455-469.

Dekker J., Rippe K., Dekker M., Kleckner N. Capturing chromosome
conformation. Science. 2002;295:1306-1311.

Diao Y., Li B., Meng Z., Jung I., Lee A.Y., Dixon J., Maliskova L.,
Guan K.L., Shen Y., Ren B. A new class of temporarily phenotypic
enhancers identified by CRISPR/Cas9-mediated genetic screening.
Genome Res. 2016;26:397-405.

Dickel D.E., Ypsilanti A.R., Pla R., Zhu Y., Barozzi I., Mannion B.J.,
Khin Y.S., Fukuda-Yuzawa Y., Plajzer-Frick I., Pickle C.S., Lee E.A.,
Harrington A.N., Pham Q.T., Garvin T.H., Kato M., Osterwalder M.,
Akiyama J.A., Afzal V., Rubenstein J.L.R., Pennacchio L.A., Visel A.
Ultraconserved enhancers are required for normal development.
Cell. 2018;172:491-499.e415.

Dickel D.E., Zhu Y., Nord A.S., Wylie J.N., Akiyama J.A., Afzal V.,
Plajzer-Frick I., Kirkpatrick A., Gottgens B., Bruneau B.G., Visel A.,
Pennacchio L.A. Function-based identification of mammalian enhancers using site-specific integration. Nat. Meth. 2014;11:566-571.

Dixon J.R., Jung I., Selvaraj S., Shen Y., Antosiewicz-Bourget J.E.,
Lee A.Y., Ye Z., Kim A., Rajagopal N., Xie W., Diao Y., Liang J.,
Zhao H., Lobanenkov V.V., Ecker J.R., Thomson J.A., Ren B. Chromatin architecture reorganization during stem cell differentiation.
Nature. 2015;518:331-336.

Dixon J.R., Selvaraj S., Yue F., Kim A., Li Y., Shen Y., Hu M., Liu J.S.,
Ren B. Topological domains in mammalian genomes identified by
analysis of chromatin interactions. Nature. 2012;485:376-380.

Ernst J., Kheradpour P., Mikkelsen T.S., Shoresh N., Ward L.D., Epstein C.B., Zhang X., Wang L., Issner R., Coyne M., Ku M., Durham T., Kellis M., Bernstein B.E. Mapping and analysis of chromatin state dynamics in nine human cell types. Nature. 2011;473:43-49.

Fang R., Yu M., Li G., Chee S., Liu T., Schmitt A.D., Ren B. Mapping
of long-range chromatin interactions by proximity ligation-assisted
ChIP-seq. Cell Res. 2016;26:1345-1348.

Farley E.K., Olson K.M., Zhang W., Brandt A.J., Rokhsar D.S.,
Levine M.S. Suboptimization of developmental enhancers. Science.
2015;350:325-328.

Forrest A.R., Kawaji H., Rehli M., Baillie J.K., de Hoon M.J., Haberle V., Lassmann T., …, Bajic V.B., Taylor M.S., Makeev V.J., Sandelin A., Hume D.A., Carninci P., Hayashizaki Y. A promoter-level
mammalian expression atlas. Nature. 2014;507:462-470.

Fullwood M.J., Ruan Y. ChIP-based methods for the identification
of long-range chromatin interactions. J. Cell. Biochem. 2009;107:
30-39.

Furlong E.E.M., Levine M. Developmental enhancers and chromosome
topology. Science. 2018;361:1341-1345.

Gasperini M., Hill A.J., McFaline-Figueroa J.L., Martin B., Kim S.,
Zhang M.D., Jackson D., Leith A., Schreiber J., Noble W.S., Trapnell C., Ahituv N., Shendure J. A genome-wide framework for mapping gene regulation via cellular genetic screens. Cell. 2019;176:
1516.

Gaulton K.J., Nammo T., Pasquali L., Simon J.M., Giresi P.G., Fogarty M.P., Panhuis T.M., Mieczkowski P., Secchi A., Bosco D., Berney T., Montanya E., Mohlke K.L., Lieb J.D., Ferrer J. A map of
open chromatin in human pancreatic islets. Nat. Genet. 2010;42:
255-259.

Gavrilov A., Eivazova E., Priozhkova I., Lipinski M., Razin S., Vassetzky Y. Chromosome conformation capture (from 3C to 5C) and
its ChIP-based modification. Methods Mol. Biol. 2009;567:171-188.

Geyer P.K., Corces V.G. DNA position-specific repression of transcription by a Drosophila zinc finger protein. Genes Dev. 1992;6:
1865-1873.

Ghavi-Helm Y., Klein F.A., Pakozdi T., Ciglar L., Noordermeer D., Huber W., Furlong E.E. Enhancer loops appear stable during development and are associated with paused polymerase. Nature. 2014; 512:
96-100.

Gomes A.L., Abeel T., Peterson M., Azizi E., Lyubetskaya A., Carvalho L., Galagan J. Decoding ChIP-seq with a double-binding signal
refines binding peaks to single-nucleotides and predicts cooperative
interaction. Genome Res. 2014;24:1686-1697.

Halfon M.S., Carmena A., Gisselbrecht S., Sackerson C.M., Jimenez F.,
Baylies M.K., Michelson A.M. Ras pathway specificity is determined by the integration of multiple signal-activated and tissuerestricted transcription factors. Cell. 2000;103:63-74.

Hammar P., Leroy P., Mahmutovic A., Marklund E.G., Berg O.G.,
Elf J. The lac repressor displays facilitated diffusion in living cells.
Science. 2012;336:1595-1598.

Harris M.B., Mostecki J., Rothman P.B. Repression of an interleukin4-responsive promoter requires cooperative BCL-6 function. J. Biol.
Chem. 2005;280:13114-13121.

Heintzman N.D., Stuart R.K., Hon G., Fu Y., Ching C.W., HawkinsR.D.,
Barrera L.O., Van Calcar S., Qu C., Ching K.A., Wang W., Weng Z.,
Green R.D., Crawford G.E., Ren B. Distinct and predictive chromatin signatures of transcriptional promoters and enhancers in the
human genome. Nat. Genet. 2007;39:311-318.

Huang D., Petrykowska H.M., Miller B.F., Elnitski L., Ovcharenko I.
Identification of human silencers by correlating cross-tissue epigenetic profiles and gene expression. Genome Res. 2019;29:657-
667.

Inoue F., Kircher M., Martin B., Cooper G.M., Witten D.M., McManus M.T., Ahituv N., Shendure J. A systematic comparison reveals
substantial differences in chromosomal versus episomal encoding of
enhancer activity. Genome Res. 2017;27:38-52.

Jung Y.L., Luquette L.J., Ho J.W., Ferrari F., Tolstorukov M., Minoda A., Issner R., Epstein C.B., Karpen G.H., Kuroda M.I., Park P.J.
Impact of sequencing depth in ChIP-seq experiments. Nucleic Acids
Res. 2014;42:e74.

Kaya-Okur H.S., Wu S.J., Codomo C.A., Pledger E.S., Bryson T.D.,
Henikoff J.G., Ahmad K., Henikoff S. CUT&Tag for efficient epigenomic profiling of small samples and single cells. Nat. Commun.
2019;10:1930.

Kel A.E., Gossling E., Reuter I., Cheremushkin E., Kel-Margoulis O.V.,
Wingender E. MATCH: A tool for searching transcription factor
binding sites in DNA sequences. Nucleic Acids Res. 2003;31:3576-
3579.

Kellis M., Wold B., Snyder M.P., Bernstein B.E., Kundaje A., Marinov G.K., Ward L.D., Birney E., Crawford G.E., Dekker J., Dunham I., Elnitski L.L., Farnham P.J., Feingold E.A., Gerstein M.,
Giddings M.C., Gilbert D.M., Gingeras T.R., Green E.D., Guigo R.,
Hubbard T., Kent J., Lieb J.D., Myers R.M., Pazin M.J., Ren B.,
Stamatoyannopoulos J.A., Weng Z., White K.P., Hardison R.C. Defining functional DNA elements in the human genome. Proc. Natl.
Acad. Sci. USA. 2014;111:6131-6138.

Kellum R., Schedl P. A position-effect assay for boundaries of higher
order chromosomal domains. Cell. 1991;64:941-950.

Kellum R., Schedl P. A group of scs elements function as domain
boundaries in an enhancer-blocking assay. Mol. Cell. Biol. 1992;12:
2424-2431.

Kheradpour P., Ernst J., Melnikov A., Rogov P., Wang L., Zhang X.,
Alston J., Mikkelsen T.S., Kellis M. Systematic dissection of regulatory motifs in 2000 predicted human enhancers using a massively
parallel reporter assay. Genome Res. 2013;23:800-811.

Kim M.J., Ahituv N. The hydrodynamic tail vein assay as a tool for the
study of liver promoters and enhancers. Methods Mol. Biol. 2013;
1015:279-289.

Kundaje A., Meuleman W., Ernst J., Bilenky M., Yen A., Heravi-Moussavi A., Kheradpour P., …, Hirst M., Meissner A., Milosavljevic A.,
Ren B., Stamatoyannopoulos J.A., Wang T., Kellis M. Integrative
analysis of 111 reference human epigenomes. Nature. 2015;518:
317-330.

Kvon E.Z., Kazmar T., Stampfel G., Yanez-Cuna J.O., Pagani M.,
Schernhuber K., Dickson B.J., Stark A. Genome-scale functional
characterization of Drosophila developmental enhancers in vivo.
Nature. 2014;512:91-95.

Kvon E.Z., Stampfel G., Yanez-Cuna J.O., Dickson B.J., Stark A. HOT
regions function as patterned developmental enhancers and have a
distinct cis-regulatory signature. Genes Dev. 2012;26:908-913.

Kwasnieski J.C., Fiore C., Chaudhari H.G., Cohen B.A. High-throughput functional testing of ENCODE segmentation predictions. Genome Res. 2014;24:1595-1602.

Kwasnieski J.C., Mogno I., Myers C.A., Corbo J.C., Cohen B.A. Complex effects of nucleotide variants in a mammalian cis-regulatory
element. Proc. Natl. Acad. Sci. USA. 2012;109:19498-19503.

Lanzuolo C., Roure V., Dekker J., Bantignies F., Orlando V. Polycomb
response elements mediate the formation of chromosome higherorder structures in the bithorax complex. Nat. Cell Biol. 2007;9:
1167-1174.

Lettice L.A., Williamson I., Devenney P.S., Kilanowski F., Dorin J.,
Hill R.E. Development of five digits is controlled by a bipartite longrange cis-regulator. Development. 2014;141:1715-1725.

Li K., Liu Y., Cao H., Zhang Y., Gu Z., Liu X., Yu A., Kaphle P., Dickerson K.E., Ni M., Xu J. Interrogation of enhancer function by
enhancer-targeting CRISPR epigenetic editing. Nat. Commun. 2020;
11:485.

Li L., He S., Sun J.M., Davie J.R. Gene regulation by Sp1 and Sp3.
Biochem. Cell Biol. 2004;82:460-471.

Lieberman-Aiden E., van Berkum N.L., Williams L., Imakaev M., Ragoczy T., Telling A., Amit I., Lajoie B.R., Sabo P.J., Dorschner M.O.,
Sandstrom R., Bernstein B., Bender M.A., Groudine M., Gnirke A.,
Stamatoyannopoulos J., Mirny L.A., Lander E.S., Dekker J. Comprehensive mapping of long-range interactions reveals folding principles of the human genome. Science. 2009;326:289-293.

Manolio T.A., Collins F.S., Cox N.J., Goldstein D.B., Hindorff L.A.,
Hunter D.J., McCarthy M.I., …, Clark A.G., Eichler E.E., Gibson G.,
Haines J.L., Mackay T.F., McCarroll S.A., Visscher P.M. Finding
the missing heritability of complex diseases. Nature. 2009;461:
747-753.

Maricque B.B., Chaudhari H.G., Cohen B.A. A massively parallel reporter assay dissects the influence of chromatin structure on cis-regulatory activity. Nat. Biotechnol. 2018. DOI 10.1038/nbt.4285.

Maricque B.B., Dougherty J.D., Cohen B.A. A genome-integrated massively parallel reporter assay reveals DNA sequence determinants
of cis-regulatory activity in neural cells. Nucleic Acids Res. 2017;
45:e16.

Maurano M.T., Humbert R., Rynes E., Thurman R.E., Haugen E.,
Wang H., Reynolds A.P., …, Ziegler S., Cotsapas C., Sotoodehnia N.,
Glass I., Sunyaev S.R., Kaul R., Stamatoyannopoulos J.A. Systematic localization of common disease-associated variation in regulatory DNA. Science. 2012;337:1190-1195.

Melnikov A., Murugan A., Zhang X., Tesileanu T., Wang L., Rogov P.,
Feizi S., Gnirke A., Callan C.G. Jr., Kinney J.B., Kellis M., Lander E.S., Mikkelsen T.S. Systematic dissection and optimization of
inducible enhancers in human cells using a massively parallel reporter assay. Nat. Biotechnol. 2012;30:271-277.

Miguel-Escalada I., Pasquali L., Ferrer J. Transcriptional enhancers:
functional insights and role in human disease. Curr. Opin. Genet.
Dev. 2015;33:71-76.

Muller H.J. Types of visible variations induced by X-rays in Drosophila. J. Genet. 1930;299-334.

Mumbach M.R., Rubin A.J., Flynn R.A., Dai C., Khavari P.A., Greenleaf W.J., Chang H.Y. HiChIP: efficient and sensitive analysis of protein-directed genome architecture. Nat. Methods. 2016;13:919-922.

Murtha M., Tokcaer-Keskin Z., Tang Z., Strino F., Chen X., Wang Y.,
Xi X., Basilico C., Brown S., Bonneau R., Kluger Y., Dailey L.
FIREWACh: high-throughput functional detection of transcriptional
regulatory modules in mammalian cells. Nat. Methods. 2014;11:
559-565.

Nagy P.L., Cleary M.L., Brown P.O., Lieb J.D. Genomewide demarcation of RNA polymerase II transcription units revealed by physical
fractionation of chromatin. Proc. Natl. Acad. Sci. USA. 2003;100:
6364-6369.

Nettling M., Treutler H., Cerquides J., Grosse I. Detecting and correcting the binding-affinity bias in ChIP-seq data using inter-species information. BMC Genomics. 2016;17:347.

Nora E.P., Goloborodko A., Valton A.L., Gibcus J.H., Uebersohn A.,
Abdennur N., Dekker J., Mirny L.A., Bruneau B.G. Targeted degradation of CTCF decouples local insulation of chromosome domains from genomic compartmentalization. Cell. 2017;169:930-
944.e922.

Pang B., Snyder M.P. Systematic identification of silencers in human
cells. Nat. Genet. 2020;52:254-263.

Paredes S.H., Melgar M.F., Sethupathy P. Promoter-proximal CCCTCfactor binding is associated with an increase in the transcriptional
pausing index. Bioinformatics. 2013;29:1485-1487.

Pasquali L., Gaulton K.J., Rodriguez-Segui S.A., Mularoni L., MiguelEscalada I., Akerman I., Tena J.J., …, Berney T., Gloyn A.L., Ravassard P., Skarmeta J.L.G., Muller F., McCarthy M.I., Ferrer J.
Pancreatic islet enhancer clusters enriched in type 2 diabetes riskassociated variants. Nat. Genet. 2014;46:136-143.

Patwardhan R.P., Hiatt J.B., Witten D.M., Kim M.J., Smith R.P.,
May D., Lee C., Andrie J.M., Lee S.I., Cooper G.M., Ahituv N., Pennacchio L.A., Shendure J. Massively parallel functional dissection
of mammalian enhancers in vivo. Nat. Biotechnol. 2012;30:265-270.

Pennacchio L.A., Bickmore W., Dean A., Nobrega M.A., Bejerano G.
Enhancers: five essential questions. Nat. Rev. Genet. 2013;14:288-
295.

Phillips-Cremins J.E., Corces V.G. Chromatin insulators: linking genome organization to cellular function. Mol. Cell. 2013;50:461-474.

Phillips-Cremins J.E., Sauria M.E., Sanyal A., Gerasimova T.I., Lajoie B.R., Bell J.S., Ong C.T., Hookway T.A., Guo C., Sun Y.,
Bland M.J., Wagstaff W., Dalton S., McDevitt T.C., Sen R., Dekker J., Taylor J., Corces V.G. Architectural protein subclasses shape
3D organization of genomes during lineage commitment. Cell.
2013;153:1281-1295.

Rada-Iglesias A., Bajpai R., Swigut T., Brugmann S.A., Flynn R.A.,
Wysocka J. A unique chromatin signature uncovers early developmental enhancers in humans. Nature. 2011;470:279-283.

Rao S.S., Huntley M.H., Durand N.C., Stamenova E.K., Bochkov I.D.,
Robinson J.T., Sanborn A.L., Machol I., Omer A.D., Lander E.S.,
Aiden E.L. A 3D map of the human genome at kilobase resolution reveals principles of chromatin looping. Cell. 2014;159:1665-
1680.

Rao S.S.P., Huang S.C., Glenn St Hilaire B., Engreitz J.M., Perez E.M.,
Kieffer-Kwon K.R., Sanborn A.L., Johnstone S.E., Bascom G.D.,
Bochkov I.D., Huang X., Shamim M.S., Shin J., Turner D., Ye Z.,
Omer A.D., Robinson J.T., Schlick T., Bernstein B.E., Casellas R.,
Lander E.S., Aiden E.L. Cohesin loss eliminates all loop domains.
Cell. 2017;171:305-320 e324.

Rye M.B., Saetrom P., Drablos F. A manually curated ChIP-seq benchmark demonstrates room for improvement in current peak-finder programs. Nucleic Acids Res. 2011;39:e25. DOI 10.1093/nar/gkq1187.

Sandmann T., Girardot C., Brehme M., Tongprasit W., Stolc V., Furlong E.E. A core transcriptional network for early mesoderm
development in Drosophila melanogaster. Genes Dev. 2007;21:
436-449.

Sanjana N.E., Wright J., Zheng K., Shalem O., Fontanillas P., Joung J.,
Cheng C., Regev A., Zhang F. High-resolution interrogation of functional elements in the noncoding genome. Science. 2016;353:1545-
1549.

Shlyueva D., Stampfel G., Stark A. Transcriptional enhancers: from
properties to genome-wide predictions. Nat. Rev. Genet. 2014;15:
272-286.

Shukla S., Kavak E., Gregory M., Imashimizu M., Shutinoski B.,
Kashlev M., Oberdoerffer P., Sandberg R., Oberdoerffer S. CTCFpromoted RNA polymerase II pausing links DNA methylation to
splicing. Nature. 2011;479:74-79.

Skene P.J., Henikoff S. An efficient targeted nuclease strategy for highresolution mapping of DNA binding sites. eLife. 2017;6:e21856.
DOI 10.7554/eLife.21856.

Song L., Crawford G.E. DNase-seq: a high-resolution technique for
mapping active gene regulatory elements across the genome from
mammalian cells. Cold Spring Harb. Protoc. 2010;2010(2):pdbprot
5384.

Song L., Zhang Z., Grasfeder L.L., Boyle A.P., Giresi P.G., Lee B.K., Crawford G.E., Lieb J.D., Furey T.S. Open chromatin defined by
DNaseI and FAIRE identifies regulatory elements that shape celltype identity. Genome Res. 2011;21:1757-1767.
Srinivasan L., Atchison M.L. YY1 DNA binding and PcG recruitment
requires CtBP. Genes Dev. 2004;18:2596-2601.

Stitzel M.L., Sethupathy P., Pearson D.S., Chines P.S., Song L., Erdos M.R., Welch R., Parker S.C., Boyle A.P., Scott L.J., Margulies E.H., Boehnke M., Furey T.S., Crawford G.E., Collins F.S.
Global epigenomic analysis of primary human pancreatic islets provides insights into type 2 diabetes susceptibility loci. Cell Metab.
2010;12:443-455.

Sur I., Taipale J. The role of enhancers in cancer. Nat. Rev. Cancer.
2016;16:483-493.

Tiwari V.K., McGarvey K.M., Licchesi J.D., Ohm J.E., Herman J.G.,
Schubeler D., Baylin S.B. PcG proteins, DNA methylation, and gene
repression by chromatin looping. PLoS Biol. 2008;6:2911-2927.

Tolhuis B., Palstra R.J., Splinter E., Grosveld F., de Laat W. Looping
and interaction between hypersensitive sites in the active beta-globin
locus. Mol. Cell. 2002;10:1453-1465.

Trynka G., Sandor C., Han B., Xu H., Stranger B.E., Liu X.S., Raychaudhuri S. Chromatin marks identify critical cell types for fine
mapping complex trait variants. Nat. Genet. 2013;45:124-130.

Visel A., Blow M.J., Li Z., Zhang T., Akiyama J.A., Holt A., PlajzerFrick I., Shoukry M., Wright C., Chen F., Afzal V., Ren B., Rubin E.M., Pennacchio L.A. ChIP-seq accurately predicts tissue-specific activity of enhancers. Nature. 2009;457:854-858.
Whyte W.A., Orlando D.A., Hnisz D., Abraham B.J., Lin C.Y.,
Kagey M.H., Rahl P.B., Lee T.I., Young R.A. Master transcription
factors and mediator establish super-enhancers at key cell identity
genes. Cell. 2013;153:307-319.

Yang J., Corces V.G. Chromatin insulators: a role in nuclear organization and gene expression. Adv. Cancer Res. 2011;110:43-76.
Yang S.H., Cheng P.H., Sullivan R.T., Thomas J.W., Chan A.W. Lentiviral integration preferences in transgenic mice. Genesis. 2008;46:
711-718.

Zabidi M.A., Arnold C.D., Schernhuber K., Pagani M., Rath M.,
Frank O., Stark A. Enhancer-core-promoter specificity separates developmental and housekeeping gene regulation. Nature. 2015;518:
556-559.

